# Factors associated with change in objectively measured physical activity in older people – data from the physical activity cohort Scotland study

**DOI:** 10.1186/s12877-017-0578-1

**Published:** 2017-08-14

**Authors:** Clare L. Clarke, Falko F. Sniehotta, Thenmalar Vadiveloo, Ishbel S. Argo, Peter T. Donnan, Marion E. T. McMurdo, Miles D. Witham

**Affiliations:** 1 School of Medicine, University of Dundee, Dundee, Scotland UK; 2 Institute of Health and Society, Newcastle University, Newcastle, England UK

**Keywords:** Physical activity, Older adults, Aging, Accelerometry, Public health

## Abstract

**Background:**

Cross-sectional relationships between physical activity and health have been explored extensively, but less is known about how physical activity changes with time in older people. The aim of this study was to assess baseline predictors of how objectively measured physical activity changes with time in older people.

**Methods:**

Longitudinal cohort study using data from the Physical Activity Cohort Scotland. A sample of community-dwelling older people aged 65 and over were recruited in 2009–2011, then followed up 2–3 years later. Physical activity was measured using Stayhealthy RT3 accelerometers over 7 days. Other data collected included baseline comorbidity, health-related quality of life (SF-36), extended Theory of Planned Behaviour Questionnaire and Social Capital Module of the General Household Survey. Associations between follow-up accelerometer counts and baseline predictors were analysed using a series of linear regression models, adjusting for baseline activity levels and follow-up time.

**Results:**

Follow up data were available for 339 of the original 584 participants. The mean age was 77 years, 185 (55%) were female and mean follow up time was 26 months. Mean activity counts fell by between 2% per year (age < =80, deprivation decile 5–10) and 12% per year (age > 80, deprivation decile 5–10) from baseline values. In univariate analysis age, sex, deprivation decile, most SF-36 domains, most measures of social connectedness, most measures from the extended Theory of Planned Behaviour, hypertension, diabetes mellitus, chronic pain and depression score were significantly associated with adjusted activity counts at follow-up. In multivariate regression age, satisfactory friend network, SF-36 physical function score, and the presence of diabetes mellitus were independent predictors of activity counts at follow up after adjustment for baseline count and duration of follow up.

**Conclusions:**

Health status and social connectedness, but not extended Theory of Planned Behaviour measures, independently predicted changes in physical activity in community dwelling older people.

**Electronic supplementary material:**

The online version of this article (doi:10.1186/s12877-017-0578-1) contains supplementary material, which is available to authorized users.

## Background

Regular physical activity is an important determinant of health and function in later life, however, most adults remain sedentary. Inactivity is particularly common amongst older people, with <10% of people over the age of 75 years in the UK reaching current physical activity recommendations [1, 2]. Physical activity promotion has often focussed on vigorous activity [3], and interventions have often targeted individual-level factors at the expense of social and environmental factors [4]. Such approaches are unlikely to be sufficient, and successful strategies to encourage physical activity in later life are likely to require complex interventions aimed at modifying several factors.

Cross-sectional relationships between physical activity, health, geography, psychology and social factors in older people have been explored extensively [5–8], and low levels of physical activity in old age predict future ill-health and earlier death [9, 10]. Less is known about how physical activity levels change in older people, what factors predict such changes, and how the trajectory of change in activity (stable, improving or declining) might affect future disability. Furthermore, many previous studies have not measured activity objectively, instead relying on subjective reports of activity, often using questionnaires. Such approaches are known to lack accuracy in older people as well as younger people [11, 12], and there is therefore a clear need for studies using objective methods of capturing how much PA older people undertake in their daily lives.

We previously established the Physical Activity Cohort Scotland (PACS) to objectively measure physical activity in a sample of nearly 600 community dwelling older people from Tayside, Scotland [5]. In this paper, we report the results of follow-up measurements of physical activity taken 2–3 years after the baseline visit in the PACS cohort, and examine the factors predicting change in objectively measured physical activity in this cohort of older, community dwelling people. We hypothesised that demographic, health, social and psychological factors would all predict changes in physical activity with time.

## Methods

### Study population and recruitment

The PACS cohort consists of 584 community-dwelling older people aged 65 and over, living in Tayside, Scotland, who were recruited to PACS between October 2009 and January 2011. The recruitment process and baseline data have been reported in detail previously [5]. Participants were recruited from 17 primary care practices; a total of 3343 invitations were sent via primary care physicians to potential participants. Sampling was stratified according to age (65–80 and 80+ years) and deprivation status (Scottish Index of Multiple Deprivation score decile 1–4 versus 5–10; http://www.isdscotland.org). Exclusion criteria for the baseline visits were: residency in institutional care, unwilling to participate, wheelchair or bedbound, the presence of cognitive impairment sufficient to prevent written informed consent or enrolment in another research study. Participants using walking aids were not excluded from the cohort.

Participants from the first wave of the PACS study were contacted between 2 and 3 years later to invite them to participate in the follow-up study. Participants were eligible for inclusion if they were aged 65 years or over and had participated in the original PACS study. Those with cognitive impairment sufficient to preclude informed consent and those unwilling to participate were excluded from participation in the second wave.

### Assessment schedule

Surviving study participants from the first wave of the PACS study who were still resident within Tayside were sent a letter of invitation. Vital status was checked against the Scottish death register prior to re-contacting participants. Interested participants were telephoned by the research nurse and an appointment made to visit them at home and undertake the assessments. The assessments took place between April 2012 and 30th January 2013. At the home visit the study was explained and informed consent obtained. The SF-36 (Health Questionnaire), extended Theory of Planned Behaviour (TPB) Questionnaire (behavioural beliefs, normative beliefs, attitude, subjective norm, self-efficacy, intention plus action planning, coping planning and social support, all on 1 to 6 scales, where 6 was strongest or highest for each domain), and the Social Capital Module of the General Household Survey were all administered at baseline [13–15]. Selected self-reported health conditions (comorbidities) were collected as part of the Older People and Active Living (OPAL) questionnaire, administered at baseline [16]. The Hospital Anxiety and Depression score (HADS) was used to collect anxiety and depression data at baseline [17]. Deprivation status at baseline was characterised by deciles of the Scottish Index of Multiple Deprivation (SIMD); this measure assigns a deprivation score to each postcode district in Scotland, based on a composite measure of employment, income, housing, crime and education. Decile 1 represents the most-deprived.

At the baseline and follow up visits, each individual was provided with an RT3 accelerometer (Stayhealthy Inc., Monrovia, California, USA) to wear on the waistband over the same hip during waking hours for a single 7-day period. The RT3 is a piezoelectric, triaxial accelerometer which has previously been validated in a number of different ways: it shows adequate test-retest reliability, it has been shown to discriminate walking from sedentary activity in older people, and it is responsive to interventions designed to increase physical activity [18–20]. Participants wore the accelerometer on the waistband anteriorly over the same hip during waking hours for a single 7-day period [20]. Summed activity counts were recorded each minute for 7 days. 24 h periods commenced at midnight; the partial data from the first and last day was therefore discarded leaving a maximum of 6 periods of 24 h for analysis. Days with less than 6 h of recorded activity data were omitted from analysis. A Freepost envelope was provided in which to return the accelerometer. Participants were instructed to remove the device at bedtime, and also not to wear the device during bathing and showering. Minutes of walking were derived from accelerometry data by counting each minute of vector-summed activity between 250 cpm and 3000 cpm. We have previously shown that these thresholds accurately classify walking in older people using the RT3 accelerometer [18]. We did not attempt to classify activity as moderate, vigorous or other based on RT3 data given the uncertainty in RT3 count thresholds for these activities in an older population.

### Data handling

Data were collated, anonymised and processed by the Health Informatics Centre, University of Dundee, according to standard operating procedures to provide data protection with Caldicott Guardian approval. A 5% random sample was entered twice to assess errors. Deaths were checked through the Health Informatics Centre (HIC) against data from the General Records Office of Scotland, which records all deaths in Scotland.

### Selection of variables for prediction modelling

We selected variables for prediction modelling based on our previous work [5], which examined potentially modifiable determinants of physical activity and showed that psychological variables, physical functioning and measures of social functioning were important associates of baseline physical activity in this population. We added measures of comorbid disease, but did not include measures of environment from baseline as these were not significantly associated with baseline activity counts in our previous analysis.

### Statistical methods

Responses to categorical questions were summarised as percentages and number of responses. Continuous variables were summarised by the mean or median if these were not normally distributed. Descriptive measures of change from baseline to follow-up were summarised as differences in means or medians, and analysis of changes between baseline and follow up were conducted using paired Student’s t-tests for normally distributed variables, or using Wilcoxon’s Signed Rank test for non-normally distributed variables.

For regression modelling, follow-up activity counts per 24 h were first adjusted for baseline activity count and length of follow up using linear regression. These adjusted activity counts were then used as the dependent variable in univariate analyses where each potential predictor was regressed against the follow-up activity count. Multivariable linear regression was then performed, using the adjusted follow-up physical activity as the dependent variable. Multiple regression using backwards elimination (*p* > 0.05 to remove) was first implemented for explanatory variables within the domains of TPB, SF-36 and Social Capital separately. Because most of the TPB measures either showed peaks at the extreme ends of the distribution, or were significantly skewed, all were recoded into high (score 4 to 6) vs low (score 1–3); the same procedure was conducted for neighbourliness in the Social Capital questionnaire. Number of people living nearby and number of people to turn to in a crisis were both skewed in distribution; both were divided into quartiles for use in the regression analyses (quartiles for number of people living nearby: 0–1, 2, 3–4, 5+; quartiles for number of people to turn to in a crisis: 0–2, 3–4, 5–6, 7+). The best candidate variables from each domain were then included in a final linear regression model (backwards elimination, *p* > 0.05 to remove), together with variables describing self-reported comorbidity derived from the baseline OPAL questionnaire. Variables with univariate *p* > 0.3 (the Hosmer-Lemeshow criterion) were not entered into multiple regression analyses. Analyses were carried out using SPSS v22 (IBM, New York, USA) and SAS v9.2 (SAS Institute, Cary, NC, USA).

## Results

30/584 people had died between the first and second surveys (5.1%); a further 23 had moved outside the Tayside geographical area and could not be contacted. Invitations were sent to 531 participants; 484/531 (91%) replied to the invitation. 361/531 (68%) agreed to participate in the follow-up survey. The mean time between baseline and follow-up assessment was 26 months (SD 4; range 17 to 37 months). 339 people participated in the follow-up survey. Those who had died were older, more likely to be male, and had lower activity counts in the baseline survey; Table 1 summarises participant characteristics, including their original accelerometry counts. A mean of 13.5 h (SD 1.8) per day of accelerometer data per participant (between rising from bed in the morning and going to bed at night) was analysable.

**Table 1:** Baseline characteristics of responders, non-responders and those who died

Variables		Whole baseline cohort	Participated in follow up	Negative response	No response	Other^a^	Dead
N (%)		584 (100)	339 (58)	123 (21)	66 (11)	26 (5)	30 (5)
Age, mean (SD)		78.5 (7.7)	77.4 (7.4)	79.9 (7.6)	78.3 (7.8)	78.0 (9.4)	85.9 (5.4)
Female sex, n (%)		317 (54)	185 (55)	68 (55)	42 (64)	9 (35)	13 (43)
SIMD	1–4, n (%)	287 (49)	147 (44)	70 (57)	37 (56)	19 (73)	14 (47)
	5–10, n (%)	293 (51)	188 (56)	53 (43)	29 (44)	7 (27)	16 (53)
Mean activity count per 24 h at baseline (SD)		146,045 (79,528)	157,407 (78,071)	128,877 (76,527)	143,442 (85,739)	144,947 (72,490)	98,795 (73,370)
Mean minutes of walking per day at baseline (SD)		177 (89)	191 (85)	155 (86)	177 (100)	172 (84)	120 (83)
Hospitalised in previous year, n (%)		99 (17)	46 (14)	25 (20)	13 (20)	6 (23)	9 (30)
Rheumatoid arthritis, n (%)		43 (7)	22 (6)	8 (7)	8 (12)	1 (4)	4 (13)
Osteoarthritis, n (%)		112 (19)	65 (19)	25 (20)	13 (20)	3 (12)	6 (20)
Neurological disease, n (%)		10 (2)	4 (1)	5 (4)	0 (0)	3 (12)	1 (3)
Hypertension, n (%)		289 (49)	161 (47)	60 (49)	38 (58)	13 (50)	17 (57)
Diabetes mellitus, n (%)		62 (11)	24 (7)	19 (15)	8 (12)	3 (12)	8 (27)
Heart disease, n (%)		186 (32)	100 (29)	41 (33)	20 (30)	8 (31)	17 (57)
Active cancer, n (%)		33 (6)	16 (5)	10 (8)	3 (5)	2 (8)	2 (7)
Fall in last year, n (%)		189 (32)	100 (29)	36 (29)	22 (33)	13 (50)	18 (60)
Presence of chronic pain, n (%)		255 (44)	143 (42)	54 (44)	31 (47)	11 (42)	16 (53)

^a^Moved away from area, plus positive replies to invitation who died or were unable to participate in follow up visits

### How do objectively measured physical activity levels change over time?

Table 2 presents changes in activity counts by strata. 309 participants had analysable accelerometry data at both baseline and follow up and are thus included in these analyses. Physical activity measured by accelerometry fell in all strata; mean activity counts fell by between 2 and 12% per year of baseline values. The mean yearly fall in activity counts was 9094 counts/24 h/year, equivalent to 6.2% per year. Both ways of analysing the accelerometry data (i.e. by activity count and by minutes of walking) produced similar results; the correlation between activity counts and minutes of walking was very high (*r* = 0.97) and thus minutes of walking are not analysed separately in regression analyses. Baseline activity counts were strongly associated with follow-up activity counts (R^2^ = 0.312); all further analyses thus used follow-up activity counts adjusted for baseline activity count and duration of follow-up.

**Table 2:** Counts and minutes of physical activity from original study (baseline) and follow up

Group	N	Activity counts/24 h – baseline mean (SD)	Activity counts/24 h - follow up, mean (SD)	Activity counts/24 h – difference between baseline and follow up		Mean difference in activity count/24 h per year		
				Mean (SD)	P*	Mean (SD)	P*	P**
A	95	168,831 (88,091)	146,764 (71,401)	−22,067 (71,392)	0.003	−10,122 (34,880)	0.006	0.73
B	35	106,829 (34,917)	89,980 (44,807)	−16,850 (46,022)	0.04	−8060 (23,282)	0.05	0.80
C	102	174,754 (73,806)	168,095 (78,802)	−6659 (78,531)	0.39	−3676 (34,303)	0.28	0.05
D	77	142,569 (74,681)	110,219 (64,763)	−32,351 (71,086)	<0.001	−15,471 (36,449)	<0.001	0.06
Group	N	Minutes walking/24 h – baseline mean (SD)	Minutes walking/24 h - follow up mean (SD)	Minutes walking/24 h – difference between baseline and follow up		Mean difference in minutes walking/24 h per year		
				Mean (SD)	P*	Mean (SD)	P*	P**
A	95	196 (85)	176 (78)	−21 (79)	0.01	−10 (41)	0.02	0.92
B	35	134 (47)	114 (57)	−18 (57)	0.07	−9 (30)	0.08	0.86
C	102	217 (86)	207 (88)	−10 (88)	0.26	−5 (40)	0.21	0.11
D	77	178 (85)	139 (88)	−38 (84)	< 0.001	−18 (42)	<0.001	0.05

Group A – SIMD 1–4 age 65–80; Group B – SIMD 1–4 age > 80; Group C - SIMD 5–10 age 65–80; Group D – SIMD 5–10 age > 80

*p for within-group change compared to baseline. **p for change compared to mean of change in other three groups

### What factors predict decline in physical activity over time in older people?

Univariate analysis (Table 3) indicated that a range of variables from the three questionnaires (social capital module, SF-36 and extended Theory of Planned Behaviour) together with age, sex, SIMD, and depression score were statistically significantly associated with adjusted follow-up daily activity counts. The highest R^2^ values were for age (0.133) and physical functioning (0.198).

**Table 3:** Univariate regression using follow-up activity count adjusted for baseline count and time to follow up as dependent variable

Variables		R^2^	Unstandardized Coefficient		t	*P* value
			B	Std Error		
Female sex		0.022	−12,598	4827	−2.01	0.01
Age (per year)		0.133	−2165	316	−6.85	<0.001
SIMD (per decile; higher decile = more affluent)		0.019	2177	894	2.44	0.015
Extended TPB (high vs low) higher score = stronger beliefs/norms/actions)	Behavioural beliefs	0.039	35,065	10,295	3.41	0.001
	Self-efficacy	0.044	19,046	5067	3.76	<0.001
	Normative belief	0.073	33,672	6956	4.84	<0.001
	Attitude	0.015	20,175	9416	2.14	0.03
	Subjective norm	0.042	7719	2105	3.67	<0.001
	Need for support	0.002	−7003	8667	−0.81	0.42
	Received support	0.005	7410	5833	1.27	0.21
	Coping planning	0.000	2828	10,674	0.27	0.79
	Action planning	0.031	15,259	4889	3.12	0.002
	Intention	0.057	22,318	5364	4.16	<0.001
SF-36 (per point; higher scores = better function)	Physical functioning	0.198	929	107	8.70	<0.001
	Role – Physical	0.037	335	97	3.45	0.001
	Bodily pain	0.031	283	91	3.10	0.002
	General health	0.072	594	121	4.90	<0.001
	Vitality	0.092	669	120	5.58	<0.001
	Social Functioning	0.030	332	108	3.08	0.002
	Role – Emotional	0.011	418	229	1.82	0.07
	Mental Health	0.028	578	196	2.95	0.003
Social Capital Module	Neighbourliness (high vs low)	0.013	12,131	6172	1.97	0.05
	Satisfactory friendship network (y vs n)	0.023	13,281	4980	2.67	0.008
	Satisfactory relative network (y vs n)	0.000	−1183	4862	−0.24	0.81
	Social support (per category)	0.012	7028	3753	1.87	0.06
	No. of people you can turn to who live nearby (per quartile)	0.017	4847	2083	2.33	0.02
	No. of people to turn to during personal crisis (per quartile)	0.019	5590	2325	2.40	0.02
Self-reported Comorbidity	Hospitalised in previous year	0.003	−6183	6958	−0.89	0.38
	Rheumatoid arthritis	0.000	−2992	9683	−0.31	0.76
	Osteoarthritis	0.006	−8398	6259	−1.34	0.18
	Neurological disease	0.002	−17,947	21,508	−0.83	0.41
	Hypertension	0.014	−9936	4845	−2.05	0.04
	Diabetes mellitus	0.027	−27,555	9543	−2.89	0.004
	Heart disease	0.004	−6211	5262	−1.18	0.24
	Active cancer	0.004	12,345	11,682	1.06	0.29
	Fall in last year	0.010	−9166	5332	−1.72	0.09
	Presence of chronic pain	0.025	−13,624	4920	−2.77	0.006
Anxiety (HADS) (per point; higher = more anxiety symptoms)		0.000	−23	751	−0.03	0.98
Depression (HADS) (per point; higher = more depressive symptoms)		0.057	−4018	931	−4.31	<0.001

SIMD: Scottish Index of Multiple Deprivation. TPB: Theory of Planned Behaviour. HADS: Hospital Anxiety and Depression Score

Multivariate regression modelling (Additional file 1: Table S1) showed that within the extended TPB, normative beliefs and action planning were independently associated with follow-up adjusted activity count (although all measures were strongly correlated with each other); within health and wellbeing, only the baseline physical function subscore of the SF-36 was independently associated with follow-up adjusted activity levels, and within the social capital model, presence, as opposed to absence of a satisfactory friend network variables was independently associated with follow-up adjusted activity levels. These factors, significant within the univariate models for TPB, SF-36 and social capital, were then entered into a combined multivariate model, with the results shown in Table 4. The retained variables (age at baseline, diabetes mellitus, satisfactory friend network and physical functioning SF-36 subscore) explained 28% of the variance in the follow-up adjusted mean activity count.

**Table 4:** Multivariate regression model (backward elimination) using adjusted follow-up activity count as dependent variable

Variables	Unstandardized Coefficient		t	*P* value
	B	Std Error		
Age at baseline (per year)	−1543	326	−4.74	<0.001
Diabetes mellitus	−22,919	8606	−2.66	0.008
Satisfactory friend network (yes vs no)	9040	4578	1.98	0.049
Physical functioning SF-36 subscore (per point)	671	120	5.60	<0.001

Variables entered into final model: Age, Sex, deprivation score, depression, osteoarthritis, hypertension, diabetes mellitus, heart disease, active cancer, fall in last year, chronic pain, physical functioning SF-36 subscore, satisfactory friend network, normative beliefs, action planning

## Discussion

### Key findings

We found that objectively measured physical activity declined in our cohort over a two year follow up period, and that older age at baseline, lower activity count at baseline, an unsatisfactory friendship network, lower self-reported physical functioning and the presence of diabetes mellitus all independently predicted a lower adjusted physical activity count at follow up. Interestingly, other self-reported comorbidities did not independently predict future physical activity levels.

### What have others found

Few studies to date have attempted to track changes in physical activity in older people over time. Data from the Rush memory and ageing project followed 519 people aged between 60 and 80, with actigraphy conducted at baseline and a mean of 6 years later. In this study, physical function declined with time (by a mean of approximately 4% per year), with steeper decreases in older people and in those with lower educational attainment. In contrast to our findings, little effect was seen for comorbid disease on the rate of decline [21]. The English Longitudinal Study of Ageing [22] examined changes in self-reported physical activity every two years over a ten year period in 5000 older people. This found a gradual decrease in the number of people reporting regular vigorous physical activity (from 35 to 26%) and a gradual increase in the proportion reporting that they were predominantly inactive (from 5% to 11%). In this study, predictors of persistent activity included younger age, male sex, not being overweight, smoking or depressed, being in work, and having no longstanding illnesses. The baseline age was much younger in this cohort (early 60’s), with comparatively little comorbid disease, hence the results may not be directly comparable with our findings.

The current literature indicates that multiple factors are associated with physical activity status in cross-sectional studies involving older people. At the individual level, these include health status and physical function, self-efficacy, mood, pet ownership and previous habits [5, 23, 24]; at the societal level factors such as social interconnectedness and neighbourliness are important [5], and at the environmental level factors such as weather (e.g. temperature and day length [25]), transport and recreational facilities are also significant [7]. Our results suggest that at least some of these factors may be important not only in explaining current levels of physical activity, but in predicting the future trajectory of physical activity. Identifying the factors that predict future activity is potentially helpful in identifying targets for intervention to maintain or enhance physical activity in older people.

Age remains a factor in our model, as with other studies discussed above. The number of birthdays an individual has celebrated per se is unlikely to influence activity levels, and thus age likely represents a constellation of unmeasured variables. Unmeasured variables that correlate strongly with age include physiological variables such as muscle strength may explain at least part of the association between age and changes in physical activity. Further work is required to unpack this constellation; measurement of physiological variables (e.g. muscle strength, fatigability) is a key aspect that we are unable to address with data from the PACS cohort. Further dissection of the social support findings is also merited – how are the effects of social contact translated into preservation of activity levels?

### Strengths and weaknesses

A key strength of our study is the substantial proportion of participants who were over the age of 80 years. The over 80s are poorly represented in studies of physical activity in older people, and there is very little evidence on how to promote physical activity for this group [8]. The use of objective measures of physical activity, repeated at a 2 year interval, has allowed us to characterise changes in activity without recall bias or the inaccuracies associated with self-reported physical activity scales [11]. Collection of a wide range of data at baseline, including psychological, environmental, health and behavioural determinants enhances our ability to provide a more complete set of predictors for future change in physical activity in older people than some previous studies.

The choice of accelerometer used to monitor physical activity in the PACS cohort has limitations. Waist-worn accelerometers cannot reliably distinguish between different postures, and soft tissue motion at the waist can induce significant errors in belt worn devices. Thus devices positioned at the waist can measure activity and inactivity patterns, but cannot measure sedentariness reliably [26]. To date, much of the validation research regarding device type, placement and data interpretation has been performed in younger, healthier populations, and translation of these methods to older populations remains problematic [27]. Although the RT3 accelerometer used in the PACS cohort has limitations, it does have the advantage of validation in older people and use in trials involving older people [18, 19]. Use of a different accelerometer, particularly one with better temporal resolution, would facilitate analysis of patterns of activity which would be likely to give greater insight than simple measuring total activity counts.

As is often the case with cohort studies with older people, there was substantial attrition between the baseline and follow-up data collection phases. We are therefore unable to describe how physical activity changed in those did not participate in the follow-up phase. This is likely to introduce a degree of bias and to limit the generalizability of our results, as those who became unwell are more likely to have dropped out. The fact that those who did not participate in the follow-up phase were older and less physically active at baseline supports this contention; those who died were much older and much less active at baseline. There are some variables that we did not collect, such as body mass index. This has previously been shown to correlate with physical activity; it is possible that the presence of diabetes as a predictor in our models reflects the effect of BMI. We have not included wear time as a covariate in our analyses; whilst short wear time may underestimate true activity levels if participants remove the accelerometer but continue activities, short wear time may also reflect periods of either rest or sleep during the daytime, or late rising / early bedtime. There is no easy way to distinguish between these possibilities using the RT3 accelerometer, and adjusting for wear time risks altering the analysis from one of total activity to one of activity per unit time, which was not the intention of this analysis.

### Translation of findings into practice

Our analysis has identified potential opportunities for designing new interventions to increase levels of physical activity in older people. Of the factors predicting future activity that we identified, two (self-reported physical function, social contact) are modifiable and the effects of one (diabetes mellitus) may be able to be mitigated. Enhancing social support in community settings is a strategy that could complement traditional approaches based on improving physical function, by capitalising on social networks to reinforce physical activity behaviour. Behavioural and social approaches including creation of ‘buddy’ systems, behavioural contracts between participants and leaders, and formation of walking or other physical activity support groups may all provide ways of operationalising such social support networks in practice. Similarly, combining existing programmes with interventions specifically targeting low mood and other mental health issues in older people could deliver ‘virtuous circles’ of improvement; improved mental health leading to enhanced physical activity, with reciprocal benefits of improved mood delivered by increased levels of physical activity and exercise [28]. Our findings pave the way for the design and appropriate targeting of novel interventions aimed at increasing physical activity participation in the most sedentary subgroup of the population – older adults. This is vital as data on barriers to physical activity in older adults are limited and interventions to improve habitual, everyday physical activity in older people (as opposed to merely providing exercise classes), have had only limited success to date. Data from analyses such as we present here will help to design a new generation of physical activity interventions, addressing both individual, societal and environmental factors together to enhance efficacy, uptake and adherence.

## Conclusion

Our study found that objectively measured physical activity declined in our cohort over a two year follow up period, and that age at baseline, activity count at baseline, satisfactory friendship network, self-reported physical functioning and the presence of diabetes mellitus all independently predicted adjusted physical activity count at follow up. Health status and social connectedness, but not extended Theory of Planned Behaviour measures, independently predicted changes in physical activity in community dwelling older people. Our analysis has identified potential targets for designing new interventions which are essential in order to increase and maintain levels of physical activity in older people.

## Additional file

Additional file 1: Tables S1. Results of multivariate regression analyses for theory of planned behaviour components, SF-36 components, and social capital module components. (DOCX 12 kb)

## Abbreviations

HADS: Hospital Anxiety and Depression Score
HIC: Health Informatics Centre
OPAL: Older People and Active Living
PACS: Physical Activity Cohort Scotland
SF-36: Short-Form 36
SIMD: Scottish Index of Multiple Deprivation
TPB: Theory of Planned Behaviour
