# Supplementary material for: Factors associated with change in objectively measured physical activity in older people – data from the physical activity cohort Scotland study
Source: BMC Geriatr. 2017 Aug 14;17:180. doi: 10.1186/s12877-017-0578-1 (PMC5557253; doi:10.1186/s12877-017-0578-1)
Supplement: Additional file 1: Tables S1. — Results of multivariate regression analyses for theory of planned behaviour components, SF-36 components, and social capital module components. (DOCX 12 kb) [file 12877_2017_578_MOESM1_ESM.docx]

**Supplementary Tables**

**Multivariate regression model predicting adjusted follow-up activity using ‘extended theory of planned behaviour’ questionnaire (backward elimination)**

| **Variables** | **Unstandardized Coefficient** | | **t** | **P value** |
| --- | --- | --- | --- | --- |
|  | **B** | **Std Error** |  |  |
| Normative beliefs (high vs low) | 24354 | 9667 | 2.52 | 0.012 |
| Action planning (high vs low) | 12901 | 5810 | 2.22 | 0.027 |

**Multivariate regression model predicting adjusted follow-up activity using SF-36 questionnaire (backward elimination)**

| **Variables** | **Unstandardized Coefficient** | | **t** | **P value** |
| --- | --- | --- | --- | --- |
|  | **B** | **Std Error** |  |  |
| Physical functioning (per point) | 930 | 109 | 8.53 | <0.001 |

**Multivariate regression model predicting adjusted follow-up activity using ‘social capital module’ questionnaire (backward elimination)**

| Variables | **Unstandardized Coefficient** | | **t** | **P value** |
| --- | --- | --- | --- | --- |
|  | **B** | **Std Error** |  |  |
| Satisfactory network of friends (yes vs no) | 14491 | 5098 | 2.84 | 0.005 |
